# Regional Differences in the Association between Dietary Patterns and Muscle Strength in Korean Older Adults: Data from the Korea National Health and Nutrition Examination Survey 2014–2016

**DOI:** 10.3390/nu12051377

**Published:** 2020-05-12

**Authors:** Ae-Rim Seo, Mi-Ji Kim, Ki-Soo Park

**Affiliations:** 1 Department of Preventive Medicine and Institute of Health Science, College of Medicine, Gyeongsang National University, Jinju 52727, Korea; sarim2101@naver.com (A.-R.S.); mijikim@gnuh.co.kr (M.-J.K.); 2 Center for Farmer’s Safety and Health, Gyeongsang National University Hospital, Jinju 52727, Korea

**Keywords:** diet, grip strength, elderly, protein

## Abstract

Objectives: Adequate nutrition is an important factor to prevent sarcopenia in older adults. The purpose of this study was to identify the regional differences in the association between dietary pattern and muscle strength in older Korean adults. Methods: This study was based on data from the Korea National Health and Nutrition Examination Survey (KNHANES) in 2014–2016. Muscle strength was measured by grip strength, and dietary patterns were derived by a cluster analysis using the k-means algorithm. Multiple logistic regression analyses were applied to determine the associations between factors (dietary patterns and residential areas) and grip strength. Results: Most participants in the rural area group (50.4%) had the Cluster Three dietary pattern (diet dominant in white rice and kimchi), while most people in the urban area group (43.8%) and the metropolitan area group (53.2%) had the Cluster One dietary pattern (diet dominant in fruits and fish). Those having poor hand grip strength represented 25.8% of the total in rural, 20.6% in urban, and 17.9% in metropolitan (*p* = 0.009) areas. Upon adjustment for socio-demographic characteristics, health behaviors, and co-morbidity, the odds ratio of Cluster Two (diet dominant in meat) was 1.601 (95%, CI: 1.001–2.563, *p* = 0.050) compared to the Cluster Three dietary pattern, but there was no significant difference in residence. Conclusions: Muscle strength in the elderly was more related to dietary pattern than was residence. Education and support for conveying the importance of protein intake in the elderly are required to motivate adequate nutrition. In addition, these actions should lead to prevention of muscle weakness and further prevent frailty.

## 1. Introduction

According to Statistics Korea, the proportion of Koreans aged 65 years or older has increased steadily and represented 14.2 percent of the Korean population in 2017 [1]. Rural areas are especially experiencing a post-aged society, with 32.5 percent of the population aged over 65 years [1]. Differences in socioeconomic and physical environment, as well as available health care resources, between rural and urban areas act to weaken the physical, mental, and social health of older adults, resulting in frailty. A recent study presented that the prevalence of frailty in Korean older adults, aged 65 or older, differs depending on residential area. Frailty prevalence was 17.4 percent in rural areas but only 10.3 percent in urban areas [2]. As the health outcome of older adults varies from rural to urban areas, it is necessary to consider regional differences when studying older adults.

Older adults experience changes in body composition with increasing age, decreasing muscle mass, and increasing body fat mass [3]. They also experience senescence with rapidly decreasing muscle strength and function at 70 years and older [3]. Sarcopenia, which involves loss of muscle mass, muscle strength, and physical function, affects frailty [4], which can result in adverse health outcomes and decrease life expectancy.

Measurement of muscle strength is relatively simple and inexpensive compared to measurement of muscle mass. Grip strength is one of the widely used methods for evaluation of myopathy in community-level epidemiological survey [5]. It is an important predictor of hospitalization, mortality, and disability [5] and a useful predictor of nutritional status [6,7]. Therefore, many researchers have used grip strength as a surrogate index of sarcopenia. 

Adequate nutrition is an important factor to prevent frailty in older adults. Because a lack of protein intake can lead to frailty, including muscle loss, bone weakness, and reduced immunity [8], a diet that contains adequate protein is recommended for older adults to prevent muscle loss.

Most studies [9,10,11,12,13,14,15] have focused not on a group of nutrients, but on individual nutrients that may help prevent sarcopenia in the older population. Since meals consist of a variety of foods with complex combinations of nutrients, rather than independent nutrients, it is necessary to consider the effect of a nutrient group or combination on sarcopenia. Recently, many studies had investigated dietary quality indicators or dietary patterns using diet-quality scores according to the recommended dietary guidelines. Previous dietary pattern studies have established the association between the Mediterranean dietary pattern and knee strength [16], as well as between healthy diet pattern and muscle protein synthesis [17]. 

However, few studies have been conducted on the relationship between dietary pattern and sarcopenia or frailty in older adults. In addition, because the conditions for purchasing food are different in urban and rural areas, there will be differences in dietary pattern between residential areas. There have been few studies on the relationship between dietary patterns and sarcopenia according to region of residence.

The purpose of this study was to identify regional differences in the association between dietary pattern and muscle strength in older Korean adults.

## 2. Subjects and Methods

### 2.1. Study Participants

**Figure 1:**
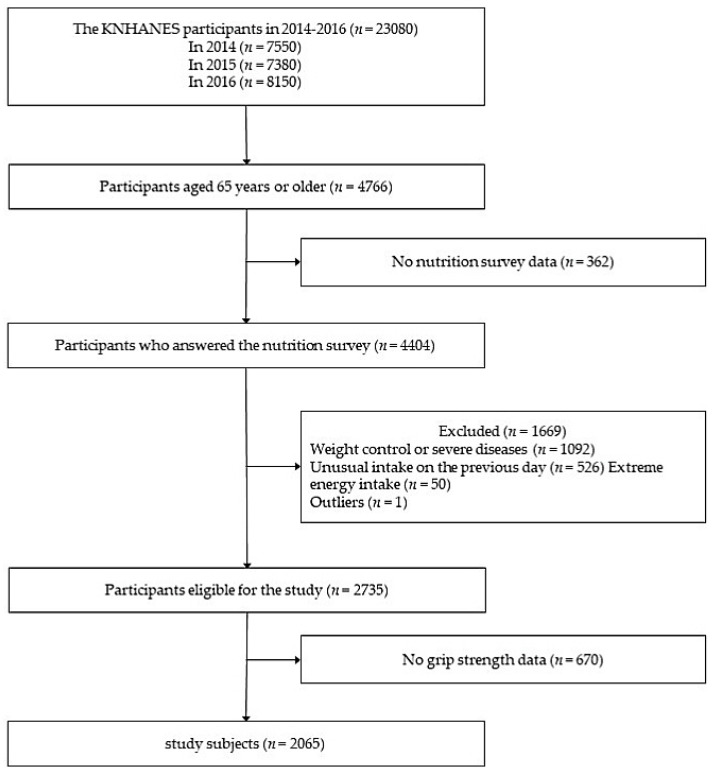
Flowchart of the study subjects.

This study was based on data from the Korea National Health and Nutrition Examination Survey (KNHANES) in 2014–2016. The KNHANES is a cross-sectional and nationally-representative survey conducted by the Korean Ministry of Health and Welfare. We recruited 4404 participants aged 65 years or older who participated in the 24-h recall method of nutrition, health, and examination survey in the KNHANES. We excluded change in eating habits due to weight control or severe diseases (*n =* 1092), unusual intake on the previous day (*n =* 526), extremely low and high energy intake (under 500 or over 5000 kcal/day, *n =* 50), missing values or outliners (*n =* 1), and unavailable grip strength data (*n =* 670). A total of 2065 participants was included in the final analyses (Figure 1). This research was approved by the Gyeongsang National University Institutional Review Board (GIRB-G19-X-0074).

### 2.2. Sociodemographic and Health-Related Characteristics

Information about sociodemographic characteristics was obtained through the health interview survey in the KNHANES. The socio-demographic characteristics were sex, age (65–74 years old and 75 years or older), education level (elementary school or less, middle school, high school, and college or more), income, marital status (living with spouse and living alone), and residential area. Income was divided into four groups: low (quartile one), low-medium (quartile two), high-medium (quartile three), and high (quartile four). Residential area was classified into three groups: metropolitan area, urban area, and rural area. Korea’s administrative districts comprise seven metropolitan cities (Seoul, Daegu, Incheon, Ulsan, Gwangju, Daejeon, and Busan). At the county level, there are *si* (cities), *gun* (counties), and *gu* (autonomous districts). These areas were further divided into *eup*, *myeon*, and *dong* at the town level. Urban areas consist of *dong* areas, and rural areas have many *eup* and *myeon* areas. In this study, the seven metropolitan cities were classified as metropolitan areas, *dong* areas as urban areas, and *eup* and *myeon* areas as rural areas. 

Information about health-related characteristics was obtained through the health interview survey and the anthropometric survey in the KNHANES. The health-related characteristics considered were muscular exercise, smoking status, high-risk drinking, history of disease, height, weight, and body mass index (BMI, kg/m^2^). High-risk drinking was defined as drinking seven glasses of beer, wine and soju at one time for men and five glasses for women and drinking more than twice per week.

### 2.3. Dietary Patterns

The KNHANES contained dietary intake data obtained by the 24-h dietary recall method. Trained investigators instructed the respondents to recall and describe all the foods and beverages they had consumed in the previous day. Dietary intake data included food name, types of ingredients, and amount of food intake by meal. The data were classified into 18 food groups according to the food code system suggested by the KNHANES.

Because grains and their products accounted for almost half of daily energy intake, this food group was further divided into five subgroups of white rice; other grains; noodles and dumpling; flour and bread; and pizza, hamburger, cereals, and snacks. Kimchi (Korean traditional fermented cabbage) was separated from other vegetables and set as a single food group because, compared to other vegetables, it is high in sodium and is eaten in high intake in Koreans. In the final analyses, we utilized a total of 22 food groups (Table S1).

The dietary patterns were derived by cluster analysis using the k-means (10 trials) algorithm. A cluster analysis aggregates individuals into relatively homogeneous subgroups (clusters) with similar dietary patterns [18]. We evaluated dietary patterns by cluster analysis using the portion size of the food consumed (g/day or mL/day), which yielded three clusters [19].

Cluster One had the highest intakes of fruits, fish and seafood, milk and dairy products and the lowest intakes of sugars and sweets, meats and meat products. We described Cluster One as a ‘diet dominant in fruits and fish’ pattern. Cluster Two had the highest intakes of beverages and alcohol, eggs, meats, and meat products and the lowest intakes of vegetables and mushrooms. We used the term ‘diet dominant in meat’ to describe this cluster. Cluster Three had the highest intakes of white rice, vegetables, and kimchi and the lowest intakes of fruits, eggs, fish and seafood. We used the term ‘diet dominant in white rice and kimchi’ pattern to describe this dietary cluster. We identified three distinct groups in the study population on cluster analyses: among them, 949 participants (45.9%) comprised Cluster One, 271 (13.4%) Cluster Two, and 845 (40.6%) Cluster Three (Table S2).

### 2.4. Muscle Strength

The KNHANES has measured grip strength with a digital hand dynamometer (digital grip strength dynamometer, T.K.K 5401, Takei Scientific Instruments Co., Ltd., Tokyo, Japan) to evaluate muscle strength since 2014. Measurements were performed three times for each hand, and the strongest result obtained with the dominant hand was recorded as the final value. Between each measurement, the individual rested at least 60 s to allow for recovery of muscle strength. Poor muscle strength based on grip strength was defined according to the Asian Working Group for Sarcopenia (AWGS) 2019 (<28 kg for men and <18 kg for women) [20]. 

### 2.5. Statistical Analysis

Statistical analyses were performed using SAS version 9.3 (SAS Institute Inc., Cary, NC, USA) and SPSS version 25.0 (IBM Corp., Armonk, NY, USA). We analyzed the KNHANES data with primary sampling units, strata, and integrated weights because it was collected using a complex sampling design involving cluster and stratified sampling. To produce the dietary patterns, we performed cluster analysis. Chi-square test for categorical variables and complex sample general linear model (CSGLM) for continuous variables were used to compare sociodemographic and health-related characteristics, dietary patterns, and grip strength of study participants according to region. Complex sample multiple logistic regression analyses (CSLOGISTIC) were applied to determine the associations between factors (dietary patterns and residential areas) and grip strength and of dietary pattern to grip strength according to region, after adjustment for sex, age, marital status, education, income, muscular exercise, smoking status, high-risk drinking, hypertension, diabetes mellitus, dyslipidemia, arthritis, height, weight, and BMI in Model Two. All tests were two-tailed with *p*-value < 0.05 considered statistically significant.

## 3. Results 

### 3.1. Sociodemographic and Health-Related Characteristics

A total of 2065 participants participated in this study. Among them, 655 people lived in rural areas, 629 in urban areas, and 781 in metropolitan areas. The sociodemographic and health-related characteristics of participants according to residential area are presented in Table 1. In the study, 980 men (49.6%) and 1085 women (50.4%) participated, and 65–74 years old was 61.1%. About one-third of the population (32.5%) was living alone. Those with an elementary school education (16.1%) and low income (27.2%) accounted for the largest percentage. Most of the population (82.5%) was not performing muscular exercise. Regarding disease history, 50.3% of the participants had hypertension, 14.9% had diabetes mellitus, 22.4% had dyslipidemia, and 24.9% had arthritis. 

There were differences in age, education, income, muscular exercise, hypertension, arthritis, height, and weight among the three residential area groups (rural, urban, and metropolitan areas) (Table 1). 

### 3.2. Dietary Patterns and Grip Strength by Residential Area

Dietary pattern and grip strength varied significantly by residential area (Table 2). Most participants in the rural area group (50.4%) had a Cluster Three dietary pattern (diet dominant in white rice and kimchi), while most people in the urban (43.8%) and metropolitan area (53.2%) groups had a cluster 1 dietary pattern (diet dominant in fruits and fish). 

Poor hand grip strength was observed in 25.8% of rural, 20.6% of urban, and 17.9% of metropolis populations (*p =* 0.009).

### 3.3. Associations between Dietary Pattern, Grip Strength, and Residential Area

The associations between dietary pattern, residence, and hand grip strength are presented in Table 3. Multiple logistic regression analysis with Model One showed an odds ratio of Cluster One of 1.995 (*p* < 0.001) and that of Cluster Two of 2.677 (*p* < 0.001) compared to the Cluster Three dietary pattern; the odds ratio of the rural population was 0.667 (*p =* 0.016) compared to the metropolitan population. Model Two, which adjusted for socio-demographic characteristics, health behaviors, and disease, yielded an odds ratio of Cluster Two of 1.601 (*p =* 0.050) compared to Cluster Three dietary pattern, but no significant difference in residence was noted. 

The relationship between dietary pattern and hand grip strength by residence is presented in Table 4. In rural areas, neither Model One or Model Two was significant; in urban areas with Model One, the odds ratio of Cluster One was 2.063 (*p* < 0001), and that of Cluster Two was 1.869 (*p =* 0.049) compared to the Cluster Three dietary pattern. In metropolitan areas with Model One, the odds ratio of Cluster One was 2.398 (*p* < 0.001) and that of Cluster Two was 8.610 (*p* < 0.001) compared to the Cluster Three dietary pattern. Model Two, which adjusted for socio-demographic characteristics, health behaviors, and disease, yielded an odds ratio of Cluster One of 1.987 (*p =* 0.016) and that of Cluster Two was 4.671 (*p =* 0.003) compared to dietary pattern Cluster Three.

## 4. Discussion

This study was performed to ascertain the relationship between dietary pattern and hand grip strength according to residence location. After adjusting for demographic variables, the group eating more meat had better muscle strength than the kimchi- and rice-based diet group, and residential location was not related to muscle strength. This tendency was evident in the metropolitan area, as the group eating a lot of fish and meat had better muscle strength.

It is difficult to accurately measure dietary intake. Further, there are many ways to obtain dietary data and extract dietary patterns, and it is not easy for dietary intake to produce the same results as factors such as culture, lifestyle, and environment [21]. However, because the 24-h recall method has an open nature, it is possible to determine various consumption patterns within a population and to compare the intakes among cultures by selecting representative samples [22]. In addition, 24-h recall does not require high cognitive ability and can provide quantitative data on food and nutrients [22], allowing wide use by the elderly.

The dietary patterns extracted from this study showed that Cluster One included a lot of fish and fruits, Cluster Two was mostly meat, and Cluster Three comprised white rice and kimchi. The dietary patterns extracted from the study of dietary patterns in the elderly were divided into the modified traditional dietary pattern (characterized by a relatively lower consumption of white rice but higher consumption of fruits, dairy products, and legumes) and the traditional dietary pattern (characterized by high consumption of white rice) [23]. The remaining dietary pattern was divided into MFDF (higher mean intakes of multigrain rice, fish, dairy products, and fruits and fruit juices) and WNC (higher mean intakes of white rice, noodles, and coffee) [24]. The white rice and kimchi-centered dietary pattern (Cluster Three) observed in this study was similar to the white rice-centered diet pattern commonly noted in previous studies and closely resembled Korean traditional diet. The Korean traditional diet contains healthy food but is high in carbohydrate intake and salt intake including kimchi, which is not necessarily a healthy food for the elderly [25]. In previous dietary patterns studies, diets related to muscle strength were classified into ’vegetables and fruits’, ’the Mediterranean diet’, ’poultry’, and the ’healthy diet’ [26]. Lack of protein intake in elderly can lead to muscle loss, bone weakness, reduced immunity, and delayed wound healing [8], while protein intake and muscle mass are positively correlated [9,27]. Protein consumption is necessary for maintaining the concentration of amino acids (particularly leucine) in the blood to increase muscle mass in the elderly [11,28], indicating need for a diet that contains enough protein for the elderly. The extracted diet patterns of Clusters One and Two contain fish and meat, both protein-based foods. To maintain elderly muscle strength, it is necessary to maintain a diet similar to that noted in Cluster One and Cluster Two containing protein, rather than Cluster Three, which was centered on white rice and kimchi. 

Compared to urban and metropolitan locations, rural areas showed higher rates of aged population, low-educated, and low-income individuals, while a diet centered on Cluster Three containing white rice and kimchi was more prevalent. Differences in dietary pattern between urban and rural areas may be due to different in food accessibility and food purchasing by residential areas. For elderly, income level was an important factor related to malnutrition. Low socioeconomic status is a known cause of malnutrition in older adults, due in part to the limited resources for accessing food. Frail older adults may become less vulnerable with strong, consistent nutritional support [29].

This study also has some limitations. First, since the KNHANES is a cross-sectional study, the association between dietary pattern and hand grip strength was confirmed, but the causal relationship cannot be clarified. Therefore, longitudinal studies are needed to confirm causality. Secondly, the nutrition data used were sourced from 24-h recall methods. This is a meal survey for the previous day and therefore might not represent an ordinary meal of the individual. However, this method is complemented by excluding outlier cases compared to an ordinary meal amount and by excluding cases influenced by diseases. Third, residence classification was based on current residence at the time of the survey and could not reflect past residence. 

In Korea, there is a rapidly increasing elderly population, which is more rapid in rural compared to urban [4]. Since muscle weakness with aging is needed to maintain health and improvements in socio-environmental factors (example: improve food accessibility in rural areas) are needed to reduce residential areas differences. In addition, education and support for the importance of protein intake in the elderly are required to ensure adequate nutrition. This action should lead to prevention of muscle weakness and to further frailty. 

## Figures and Tables

**Table 1:** Sociodemographic and health-related characteristics by residential areas.

Characteristics		Total (*n* = 2065)	Rural Area (*n* = 655)	Urban Area (*n* = 629)	Metropolitan Area (*n* = 781)	*p* Value
Gender	Men	980 (49.6)	301 (48.0)	293 (48.1)	386 (51.8)	0.386
	Women	1085 (50.4)	354 (52.0)	336 (51.9)	395 (48.2)	
Age	65–74	1230 (61.1)	349 (53.3)	372 (59.7)	509 (66.9)	<0.001
	≥75	835 (38.9)	306 (46.7)	257 (40.3)	272 (33.1)	
Marital status	Living alone	689 (32.5)	212 (32.7)	217 (33.1)	260 (32.0)	0.921
	Living together	1375 (67.5)	442 (67.3)	412 (66.9)	521 (68.0)	
Education	≤Elementary school	1155 (61.1)	417 (72.0)	353 (62.0)	385 (53.9)	<0.001
	Middle school	248 (13.2)	70 (11.7)	68 (11.8)	110 (15.0)	
	High school	270 (15.5)	62 (12.2)	94 (16.4)	114 (16.8)	
	≥College	171 (10.2)	20 (4.1)	57 (9.8)	94 (14.3)	
Income	Low (quartile 1)	552 (27.2)	234 (37.1)	139 (23.0)	179 (24.1)	<0.001
	Low-medium (quartile 2)	525 (24.4)	182 (28.7)	155 (23.3)	188 (22.4)	
	High-medium (quartile 3)	485 (22.3)	128 (18.1)	166 (25.7)	191 (22.5)	
	High (quartile 4)	487 (26.1)	106(16.1)	166 (28.0)	215 (31.0)	
Muscular exercise	<2 per week	1541 (82.5)	520 (90.8)	472 (82.5)	549 (77.3)	<0.001
	≥2 days per week	305 (17.5)	52 (9.2)	99 (17.5)	154 (22.7)	
Smoking status	Current smoking	191 (11.3)	49 (9.1)	68 (12.7)	74 (11.5)	0.318
	Past/non smoking	1719 (88.7)	547 (90.9)	515 (87.3)	657(88.5)	
High-risk drinking	No	1833 (95.7)	575 (96.4)	567 (96.8)	691 (94.4)	0.146
	Yes	81 (4.3)	22 (3.6)	18 (3.2)	41 (5.6)	
Hypertension	No	958 (49.7)	312 (51.1)	280 (46.6)	366 (51.1)	0.012
	Yes	956 (50.3)	286 (48.9)	310 (53.4)	360 (48.9)	
Diabetes mellitus	No	1636 (85.1)	525 (87.8)	493 (82.3)	618 (85.4)	0.113
	Yes	276 (14.9)	72 (12.2)	96 (17.7)	108 (14.6)	
Dyslipidemia	No	1496 (77.6)	504 (82.5)	463 (78.6)	529 (73.8)	0.492
	Yes	418 (22.4)	94 (17.5)	127 (21.4)	197 (26.2)	
Arthritis	No	1386 (75.1)	432 (72.9)	427 (75.6)	527 (76.0)	<0.001
	Yes	482 (24.9)	151 (27.1)	149 (24.4)	182 (24.0)	
Body Mass Index (m^2^/kg), mean ± SD		23.7 ± 0.1	23.6 ± 0.2	24.0 ± 0.2	23.7 ± 0.1	0.142

Values are presented as numbers (%). *p* values were determined by chi-square test and complex sample general linear model (CSGLM).

**Table 2:** Dietary patterns and grip strength by residential areas.

Factors		Total (*n* = 2065)	Rural Area (*n* = 655)	Urban Area (*n* = 629)	Metropolitan Area (*n* = 781)	*p* Value
Dietary patterns	Cluster One	949 (45.9)	243 (36.7)	294 (43.8)	412 (53.2)	<0.001
	Cluster Two	271 (13.4)	80 (12.9)	91 (14.7)	100 (12.9)	
	Cluster Three	845 (40.6)	332 (50.4)	244 (41.5)	269 (33.9)	
Grip strength	Poor	500 (24.0)	186 (29.1)	158 (25.6)	156 (19.8)	0.009
	Robust	1565 (76.0)	469 (70.9)	471 (74.4)	625 (80.2)	

Values are presented as numbers (%). *p* values were determined by chi-square test. Cluster One: diet dominant in fruits and fish; Cluster Two: diet dominant in meats; Cluster Three: diet dominant in white rice and kimchi.

**Table 3:** Factors affecting grip strength.

Factors	Model One				Model Two			
	Exp (B)	95.0% CI for Exp (B)		*p* Value	Exp (B)	95.0% CI for Exp (B)		*p* Value
		Lower	Upper			Lower	Upper	
Dietary pattern (reference: Cluster Three)								
Cluster One	1.995	1.542	2.581	<0.001	1.356	0.972	1.893	0.073
Cluster Two	2.677	1.816	3.944	<0.001	1.601	1.000	2.563	0.050
Residential area (reference: Metropolitan)								
Rural	0.667	0.480	0.926	0.016	1.004	0.680	1.482	0.984
Urban	0.748	0.540	1.037	0.081	0.877	0.601	1.282	0.498

CI: confidence interval. *p* values were determined by complex sample multiple logistic regression analysis. Cluster One: diet dominant in fruits and fish; Cluster Two: diet dominant in meats; Cluster Three: diet dominant in white rice and kimchi. Model One was unadjusted, but, involved of dietary pattern and residential area together. Model Two was adjusted for gender (men = 1, women = 0), age (continuous), marital status (living alone = 1, living together = 0), education (≤elementary school = 3, middle school = 2, high school = 1, ≥college = 0), income (low = 3, low-medium = 2, high-medium = 1, high = 0), muscular exercise (no = 1, yes = 0), smoking status (current smoking = 1, past / non-smoking = 0), high-risk drinking (no = 1, yes = 0), hypertension (no = 1, yes = 0), diabetes mellitus (no = 1, yes = 0), dyslipidemia (no = 1, yes = 0), arthritis (no = 1, yes = 0), and body mass index (continuous).

**Table 4:** Factors affecting grip strength by residential areas.

Factors		Grip Strength (Reference: Poor)											
		Rural area				Urban Area				Metropolitan Area			
		Exp (B)	95.0% CI for Exp (B)		*p* Value	Exp (B)	95.0% CI for Exp (B)		*p* Value	Exp (B)	95.0% CI for Exp (B)		*p* Value
			Lower	Upper			Lower	Upper			Lower	Upper	
Model One	Dietary pattern (reference: Cluster Three)												
	Cluster One	1.555	0.963	2.510	0.071	2.063	1.349	3.156	0.001	2.398	1.572	3.659	<0.001
	Cluster Two	1.668	0.914	3.044	0.096	1.869	1.004	3.478	0.049	8.610	3.738	19.835	<0.001
Model Two	Dietary pattern (reference: Cluster Three)												
	Cluster One	0.948	0.544	1.650	0.849	1.248	0.709	2.197	0.441	1.987	1.135	3.479	0.016
	Cluster Two	0.994	0.444	2.227	0.989	1.035	0.506	2.119	0.925	4.671	1.701	12.827	0.003

CI: confidence interval. P values were determined by complex sample multiple logistic regression analysis. Cluster One: diet dominant in fruits and fish; Cluster Two: diet dominant in meats; Cluster Three: diet dominant in white rice and kimchi. Model One was unadjusted. Model Two was adjusted for gender (men = 1, women = 0), age (continuous), marital status (living alone = 1, living together = 0), education (≤ elementary school = 3, middle school = 2, high school = 1, ≥ college = 0), income (low = 3, low-medium = 2, high-medium = 1, high = 0), muscular exercise (no = 1, yes = 0), smoking status (current smoking = 1, past / non-smoking = 0), high-risk drinking (no = 1, yes = 0), hypertension (no = 1, yes = 0), diabetes mellitus (no = 1, yes = 0), dyslipidemia (no = 1, yes = 0), arthritis (no = 1, yes = 0), and body mass index (continuous).

## Supplementary Materials

The following are available online at https://www.mdpi.com/2072-6643/12/5/1377/s1, Table S1: Food groups and items used in the dietary pattern analysis, Table S2: Intakes of food groups (g/day or mL/day^§^) by cluster.
