# Supplementary material for: Regional Differences in the Association between Dietary Patterns and Muscle Strength in Korean Older Adults: Data from the Korea National Health and Nutrition Examination Survey 2014–2016
Source: Nutrients. 2020 May 12;12(5):1377. doi: 10.3390/nu12051377 (PMC7284570; doi:10.3390/nu12051377)
Supplement: Supplementary file 1 [file nutrients-12-01377-s001.pdf]

Table S1. Food groups and items used in the dietary pattern analysis

Food group	Food items
White rice	White rice
Other grain	Brown rice, barley, millet, maize, etc.
Noodles and dumpling	Noodle, ramen, somyon, udong, dumpling, etc.
Flour and bread	Wheat flour, breads, doughnuts, cakes, etc.
Pizza, hamburger, cereals and snack	Pizza, hamburger, sandwich, cereal, cracker, snack, cookie, popcorn, biscuits, chip, etc.
Potatoes	Potato, french fried potato, sweet potato, etc.
Sugars and sweets	White sugar, honey, candy, chocolates, jelly, caramel, etc.
Legumes	Soybean, curd, green peas, red bean, etc.
Nuts and seeds	Peanuts, almonds, walnut, sunflower seed, etc.
Vegetables	All kinds of vegetables, etc.
Kimchi	All kinds of kimchi, etc.
Mushrooms	All kinds of mushroom, etc.
Fruits	Citrus fruit, strawberry, melon, banana, peach, orange, fruit juices, etc.
Meats and meat products	Beef, pork, ham, sausage, meatball, bacon, chicken, turkey, etc.
Eggs	Egg, cooked egg, fried egg, etc.
Fishes and seafood	Mackerel, bastard halibut, flat fish, canned tuna, anchovy, squid, etc.
Seaweeds	Seaweed, kelp, etc.
Milk and dairy products	Milk, ice cream, yoghurt, cheese, etc.
Oils	Olive oil, corn oil, sesame oil, etc.
Beverages and alcohol	Coffee, green tea, black tea, hot chocolate, carbonated beverage, beer, liquor, whiskey, etc.
Seasonings	Soy sauce, red pepper, mayonnaise, ketchup, salt, etc.
Etc.	Etc.

Table S2. Intakes of food groups (g /day or ml/day[§]) by cluster, n=2,065

Food group	Cluster 1 – Diet dominant in fruits and fish (n=949)	Cluster 2 – Diet dominant in meats (n=271)	Cluster 3 – Diet dominant in white rice and kimchi (n=845)
White rice	13.61	12.63	31.35
Other grain	3.68	3.28	3.78
Noodles and dumpling	2.03	2.44	1.94
Flour and bread	1.04	1.35	0.77
Pizza, hamburger, cereals and snack	0.20	0.29	0.18
Potatoes	3.35	2.56	2.06
Sugars and sweets	0.66	0.71	0.71
Legumes	2.78	2.84	4.83
Nuts and seeds	0.42	0.37	0.34
Vegetables	14.62	13.89	17.48
Kimchi	8.01	7.32	14.57
Mushrooms	0.22	0.22	0.27
Fruits	23.88	5.26	3.63
Meats and meat products	3.46	4.22	3.59
Eggs	1.23	1.34	1.07
Fishes and seafood	8.22	5.52	3.97
Seaweeds	3.08	1.13	0.86
Milk and dairy products[§]	4.69	2.72	3.03
Oils[§]	0.33	0.40	0.37
Beverages and alcohol[§]	2.31	29.44	2.40
Seasonings[§]	1.73	1.79	2.45
Etc.	0.45	0.46	0.33
